# Impact of age on treatment response in men with prostate cancer treated with radiotherapy

**DOI:** 10.1002/bco2.132

**Published:** 2021-12-27

**Authors:** Alex K. Bryant, Tyler J. Nelson, Rana R. McKay, A. Karim Kader, J. Kellogg Parsons, John P. Einck, Christopher J. Kane, Ajay P. Sandhu, Arno J. Mundt, James D. Murphy, Brent S. Rose

**Affiliations:** ^1^ Department of Radiation Oncology University of Michigan Ann Arbor Michigan USA; ^2^ Department of Radiation Medicine and Applied Sciences University of California San Diego La Jolla California USA; ^3^ Division of Hematology‐Oncology, Department of Internal Medicine University of California San Diego La Jolla California USA; ^4^ Department of Urology University of California San Diego La Jolla California USA; ^5^ Clinical and Translational Research Institute University of California San Diego La Jolla California USA; ^6^ Department of Radiation Oncology Veterans Affairs Ann Arbor Healthcare System Ann Arbor Michigan USA; ^7^ Veterans Affairs San Diego Healthcare System La Jolla California USA

**Keywords:** hormone receptor agonists, neoadjuvant therapy, prostatic neoplasm, radiotherapy, veterans

## Abstract

**Objective:**

To analyse the effect of age at diagnosis on clinical outcomes of localized prostate cancer (PCa) treated with radiation therapy.

**Subjects and methods:**

We identified 12 784 patients with intermediate‐ or high‐risk localized PCa treated with radiation therapy (RT) and neoadjuvant androgen deprivation therapy (ADT) between 2000 and 2015 from nationwide Veterans Affairs data. Patients were grouped into three age categories (≤59, 60–69, and ≥70 years old). Outcomes included immediate PSA response (3‐month post‐RT PSA and 2‐year PSA nadir, grouped into <0.10 ng/ml, 0.10–0.49 ng/ml, and ≥0.50 ng/ml), biochemical recurrence, and PCa‐specific mortality. Multivariable regression models included ordinal logistic regression for short‐term PSA outcomes, Cox regression for biochemical recurrence, and Fine‐Gray competing risks regression for PCa‐specific mortality.

**Results:**

A total of 2136 patients (17%) were ≤59 years old at diagnosis, 6107 (48%) were 60–69 years old, and 4541 (36%) were ≥70 years old. Median follow‐up was 6.3 years. Younger age was associated with greater odds of higher 3‐month PSA group (≤59 vs. ≥70: adjusted odds ratio [aOR] 1.90, 95% CI 1.64–2.20; *p* < 0.001) and higher 2‐year PSA nadir group (≤59 vs. ≥70: aOR 1.89, 95% CI 1.62–2.19, *p* < 0.001). Younger age was associated with greater risk of biochemical recurrence (≤59 vs. ≥70: adjusted hazard ratio 1.45, 95% CI 1.26–1.67, *p* < 0.001) but not PCa‐specific mortality (*p* = 0.16).

**Conclusion:**

In a large nationwide sample of US veterans treated with ADT and RT for localized PCa, younger age was associated with inferior short‐term PSA response and higher risk of biochemical recurrence.

## 1 INTRODUCTION

The influence of age at diagnosis on prostate cancer outcomes is unclear. While older patients tend to present with higher‐grade disease^1^, ^2^ and are less likely to undergo local treatment,^1^ retrospective series have differed on whether prostate cancer‐specific survival differs between younger and older patients after adjustment for clinical variables.^1^, ^2^, ^3^, ^4^, ^5^, ^6^ Some studies have also suggested that younger patients are at higher risk for biochemical failure and metastasis^4^, ^7^, ^8^, ^9^ and young age is included as a negative prognostic factor in the recently‐proposed STAR‐CAP staging system,^10^ though the biologic basis for this effect is unknown and several studies have suggested no differences.^11^, ^12^, ^13^ It is also unknown whether age influences short‐term prostate‐specific antigen (PSA) response and post‐treatment PSA nadir. It has been suggested that prostate cancer in young men may harbour mutations that predispose to more aggressive disease; age‐related declines in serum testosterone might also affect responsiveness to androgen deprivation therapy (ADT), though some studies have actually suggested poorer outcomes with a lower baseline testosterone.^14^ In this study, we leverage a large national Veterans Affairs database including patients with localized prostate cancer treated with ADT and radiation therapy (RT) to examine the effect of age at diagnosis on short‐term PSA responses, biochemical failure, and prostate cancer‐specific mortality.

## 2 SUBJECTS AND METHODS

### 2.1 Data source

We identified prostate cancer patients from the VA Informatics and Computing Infrastructure (VINCI). VINCI is a comprehensive informatics platform that allows researchers access to patient‐level electronic health record information and administrative data for all veterans within the Veterans Affairs (VA) health care system. VINCI incorporates tumour registry data uploaded from individual VA sites; these data are gathered at individual VA medical centres by trained registrars according to standard protocols issued from the American College of Surgeons. These data include veterans who are treated at non‐VA facilities if they received any care at a VA facility over the course of their illness.

### 2.2 Patient cohort

**FIGURE 1:**
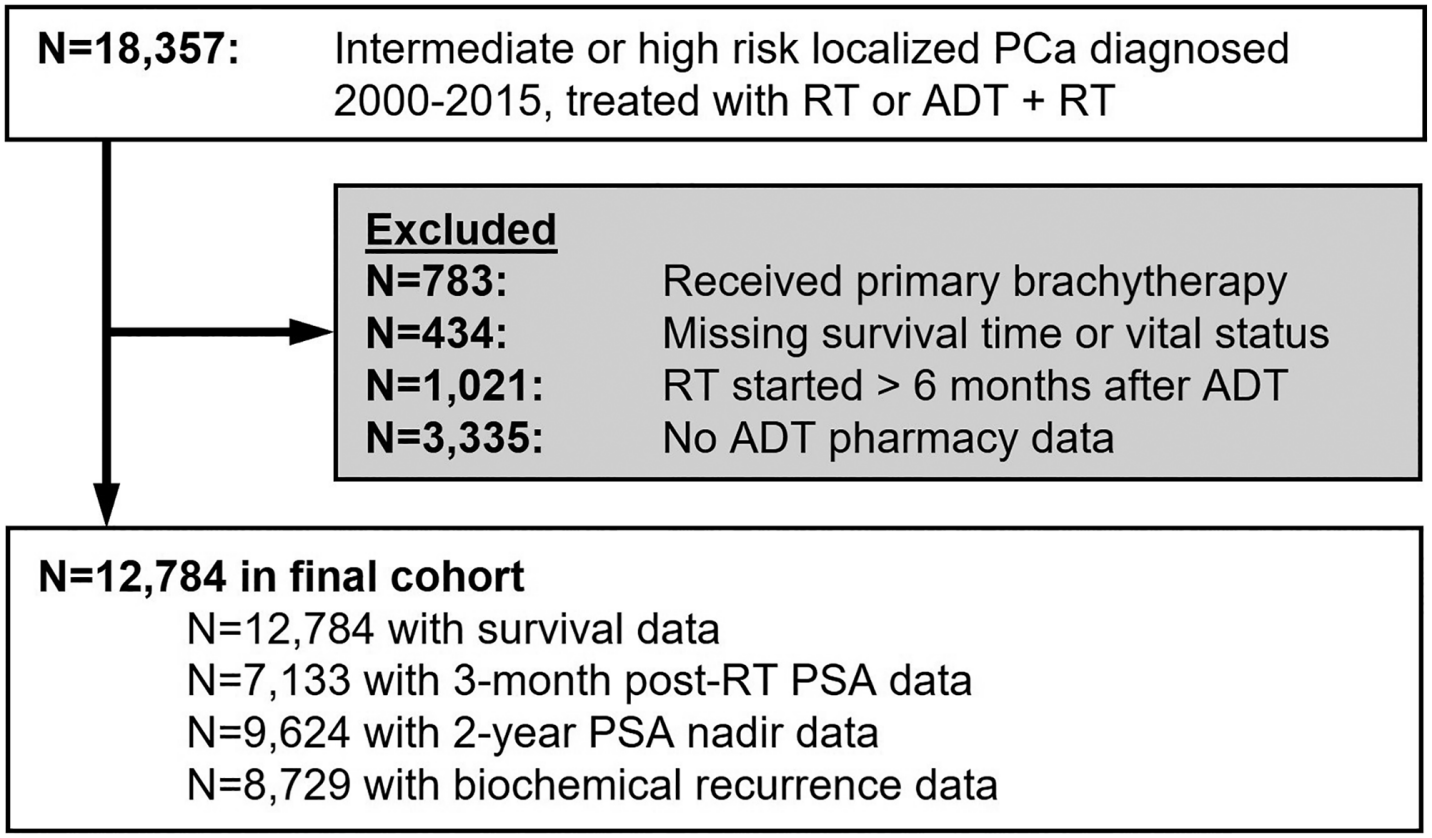
Patient selection diagram. Abbreviations: ADT, androgen deprivation therapy; PCa, prostate cancer; PSA, prostate‐specific antigen; RT, radiation therapy

The cohort included United States veterans diagnosed with intermediate‐ or high‐risk localized prostate adenocarcinoma treated with upfront neoadjuvant ADT followed by RT between 2000 and 2015. Treatment with ADT and RT was ascertained through tumour registry data. All patients on ADT were treated with gonadotropin‐releasing hormone (GNRH) agonists with or without concomitant androgen receptor antagonists. Intermediate risk prostate cancer was defined as clinical tumour stage 2b or 2c, Gleason score 7, or pre‐treatment PSA between 10 and 20 ng/ml. High risk was defined as tumour stage 3a or 3b, Gleason score 8–10, or pre‐treatment PSA greater than 20 ng/ml. We excluded patients who received primary brachytherapy, had missing survival or VA pharmacy data, or started RT greater than 6 months after starting ADT. Figure 1 shows the patient selection criteria and exclusions.

### 2.3 Outcomes

Outcomes include post‐treatment PSA response (3‐month post‐RT PSA, 2‐year PSA nadir), biochemical recurrence, and prostate cancer‐specific mortality (PCSM). Figure 1 shows the number of patients available for each analysis. For 3‐month post‐RT PSA, we searched for the PSA measurement closest to 3 months after the conclusion of RT; we included measurements within a ±2 month window of this time point.^15^, ^16^ Two‐year PSA nadir was defined as the lowest PSA measurement within 2 years after the beginning of RT. Three‐month post‐RT and 2‐year PSA nadir were grouped into <0.1 ng/ml, 0.1 to 0.49 ng/ml, and ≥0.5 ng/ml.^15^, ^16^, ^17^ Biochemical recurrence was defined as a PSA greater than or equal to the 2‐year nadir PSA + 2 ng/ml^18^ and was measured from the start of RT. For PCSM, vital status and ICD‐10 death certificate cause of death codes were obtained primarily through the National Death Index (91% of cause of death data) with missing data supplemented by the tumour registry (9%).

### 2.4 Covariates

Covariates derived from tumour registry data included age at diagnosis, clinical tumour stage, race, year of diagnosis, Gleason score, brachytherapy boost, employment, marital status, and body mass index. Age at diagnosis was classified into three age groups: ≤59, 60–69, and ≥70 years. Zip code‐level education and median income data were obtained through the 2015 American Community Survey 5‐year estimates. ADT type and anti‐androgen use were obtained from VA pharmacy records. Pre‐treatment PSA was obtained through VA laboratory data. Comorbidity was assessed with the Charlson comorbidity index and included comorbid conditions in the year prior to diagnosis.^19^

### 2.5 Statistical analysis

Baseline covariate data were compared between age groups using the chi‐square test to compare proportions, *t* test to compare means for normally‐distributed variables, and Wilcoxon rank‐sum test to compare medians for skewed variables. The association between age group and PSA response (3‐month post‐RT PSA and 2‐year PSA nadir) was assessed with univariable and multivariable ordinal logistic regression. PSA outcomes were ordered as [<0.1 ng/ml, 0.1 to 0.49 ng/ml, ≥0.5 ng/ml], and covariate effects represent the odds of membership in a higher PSA group (corresponding to inferior PSA response). For time‐to‐event outcomes, multivariable models included Cox regression for biochemical recurrence and Fine‐Gray competing risk regression for PCSM to account for the competing risk of non‐prostate cancer mortality. Survival time was measured from the start of RT. We censored patients at the last known PSA measurement for biochemical recurrence and at the last follow‐up with a VA provider for PCSM.

All multivariable models adjusted for age group, clinical tumour stage, Gleason score, pre‐treatment PSA (log‐transformed), African‐American race, Charlson comorbidity index, body mass index, year of diagnosis, median zip‐code income and education, brachytherapy boost, and anti‐androgen therapy. We used multiple imputation by fully conditional specification (FCS) to impute missing data for Gleason score (7.75% missing), clinical tumour stage (4.25%), pre‐treatment PSA (24.1%), body mass index (0.44%), median zip‐code income (2.32%), and median zip‐code education (1.91%). Continuous variables were imputed by predictive mean matching and categorical variables were imputed by multinomial logistic regression. Variables were included in imputation models if their Pearson correlation with the imputed variable was ≥0.05; the number of predictors per imputed variable ranged from 2 (for median income) to 10 (for body mass index). Convergence was assessed by visual inspection of the mean and variance of each imputed variable across 10 iterations of the FCS algorithm. Parameter estimates for statistical models were pooled across five imputed datasets by Rubin's rules. All statistical tests were two‐sided. Statistical analyses were performed with SAS v9.4 (SAS Institute, Cary, NC) and R v1.1.4 (R Core Team, Vienna, Austria).

## 3 RESULTS

### 3.1 Patient characteristics

The sample included 12 784 patients, of whom 2136 (16.7%) were ≤59 years of age at diagnosis, 6107 (47.8%) were 60–69 years, and 4541 (35.5%) were ≥70 years. Younger patients tended to have higher BMI, African‐American race, unmarried status, and live in lower income zip codes (Table 1). Younger patients were also less likely to be diagnosed with Gleason 8–10 disease and cT3 tumours. After multiple imputation of missing PSA, Gleason score, and clinical T stage data, 56.5% of the sample had high risk disease (95% CI 55.5%–57.5%). We noted no statistically significant difference in median ADT duration across age groups (≤59: 6.18 months; 60–69: 6.27 months; ≥70: 6.51 months; *p* = 0.11).

**TABLE 1:** Characteristics of the sample

Covariate		Age group			
		≤59	60–69	≥70	*p* value
Sample size, *n*		2136	6107	4541	
Age at diagnosis in years, mean (SD)		55.7 (3.13)	64.5 (2.74)	74.5 (3.53)	<0.001
Body mass index, mg/kg^2^, mean (SD)		30.2 (7.17)	29.6 (6.60)	27.4 (5.62)	<0.001
	Missing, *n* (%)	14 (0.66)	27 (0.44)	16 (0.35)	
Year of diagnosis, *n* (%)	2000–2003	304 (14.2)	797 (13.1)	911 (20.1)	<0.001
	2004–2007	639 (29.9)	1272 (20.8)	1464 (32.2)	
	2008–2011	741 (34.7)	2240 (36.7)	1354 (29.8)	
	2012–2015	452 (21.2)	1798 (29.4)	812 (17.9)	
Race	African‐American	1133 (53.0)	1959 (32.1)	1104 (24.3)	<0.001
	White	964 (45.1)	3992 (65.4)	3310 (72.9)	
	Other	39 (1.83)	156 (2.55)	127 (2.80)	
Employed full‐time, *n* (%)		331 (15.5)	651 (10.7)	130 (2.86)	<0.001
Married, *n* (%)		805 (37.7)	2903 (47.5)	2387 (52.6)	<0.001
Zip code median income, in $1000 (IQR)		43.0 (34.6–54.8)	45.8 (36.6–58.6)	46.9 (37.5–61.2)	<0.001
	Missing, *n* (%)	64 (3.00)	133 (2.18)	98 (2.16)	
Zip code % with high school diploma, median (IQR)		85.8 (80.4–90.4)	86.6 (80.6–91.4)	86.9 (80.7–91.8)	<0.001
	Missing, *n* (%)	56 (2.62)	105 (1.72)	82 (1.80)	
Charlson comorbidity index, *n* (%)	0	1298 (60.8)	3077 (50.4)	2277 (50.1)	<0.001
	1	498 (23.3)	1740 (28.5)	1303 (28.7)	
	≥2	340 (15.9)	1290 (21.1)	961 (21.2)	
Pre‐treatment PSA, ng/ml, median (IQR)		10.7 (6.10–21.7)	8.94 (5.65–16.6)	10.4 (6.30–18.0)	<0.001
	Missing, *n* (%)	509 (23.8)	1505 (24.6)	1058 (23.3)	
Gleason score, *n* (%)	6	147 (7.36)	341 (5.94)	240 (5.91)	<0.001
	7	1126 (56.4)	2961 (51.6)	1830 (45.0)	
	8–10	725 (36.3)	2435 (42.4)	1993 (49.1)	
	Missing, *n* (%)	138 (6.46)	370 (6.06)	478 (10.5)	
Tumour stage, *n* (%)	T1c‐T2a	1396 (68.2)	3981 (67.8)	2788 (64.5)	0.001
	T2b‐T2c	538 (26.3)	1486 (25.3)	1221 (28.2)	
	T3	113 (5.52)	404 (6.88)	316 (7.31)	
	Missing, *n* (%)	89 (4.17)	236 (3.86)	216 (4.76)	
Anti‐androgens, *n* (%)^a^		1147 (53.7)	3132 (51.3)	2597 (57.2)	<0.001
Brachytherapy boost, *n* (%)		79 (3.7)	186 (3.1)	96 (2.1)	<0.001
ADT duration, median in months (IQR)		6.18 (3.55–14.4)	6.27 (3.88–15.2)	6.51 (3.52–16.0)	0.12

*Note*: Percentages and *p* values are calculated based on patients with non‐missing data.

Abbreviations: ADT, androgen deprivation therapy; IQR, interquartile range; PSA, prostate‐specific antigen; SD, standard deviation.

^a^ Calculated among patients who received neoadjuvant ADT.

### 3.2 Effect of age on PSA response

Younger patients tended to have higher 3‐month post‐RT PSA (percent in ≥0.5 ng/ml group: ≤59: 16.4%; 60–69: 11.2%; ≥70: 7.91%; unadjusted *p* < 0.001) and higher 2‐year nadir PSA (percent in ≥0.5 ng/ml group: ≤59: 7.20%; 60–69: 4.25%; ≥70: 3.22%; unadjusted *p* < 0.001). In the ordinal multivariable regression model for 3‐month post‐RT PSA, younger age group was associated with increased odds of higher PSA group (≤59 vs. ≥70: OR 1.90 [95% CI 1.64–2.20], *p* < 0.001; 60–69 vs. ≥70: OR 1.37 [95% CI 1.22–1.53], *p* < 0.001; Table 2). Similar results were found in the multivariable model for 2‐year PSA nadir (≤59 vs. ≥70: OR 1.89 [95% CI 1.62–2.20], *p* < 0.001; 60–69 vs. ≥70: OR 1.36 [95% CI 1.21–1.54], *p* < 0.001; Table 2). Other predictors of inferior PSA response were largely consistent across both models and included higher pre‐treatment PSA, African‐American race, and lack of anti‐androgen therapy. Brachytherapy boost was associated with lower 3‐month post‐RT PSA but was not associated with 2‐year PSA nadir.

**TABLE 2:** Ordinal regression results for PSA response outcomes

Covariate		3‐month post‐RT PSA		2‐year PSA nadir	
		OR (95% CI)	*p* value	OR (95% CI)	*p* value
Age group	≥70	(ref)		(ref)	
	60–69	1.37 (1.22–1.53)	<0.001	1.36 (1.21–1.54)	<0.001
	≤59	1.90 (1.64–2.20)	<0.001	1.89 (1.62–2.19)	<0.001
Clinical tumour stage	1C‐2A	(ref)		(ref)	
	2B‐2C	1.13 (1.00–1.27)	0.04	1.07 (0.95–1.21)	0.29
	3	1.24 (1.01–1.53)	0.04	0.98 (0.79–1.22)	0.86
Gleason score	6	(ref)		(ref)	
	7	1.23 (1.01–1.50)	0.04	1.17 (0.95–1.44)	0.14
	8–10	1.25 (1.02–1.53)	0.04	0.80 (0.64–1.00)	0.06
ln (pre‐treatment PSA)		1.83 (1.72–1.96)	<0.001	1.52 (1.43–1.63)	<0.001
Anti‐androgen therapy		0.63 (0.57–0.69)	<0.001	0.76 (0.68–0.84)	<0.001
Charlson comorbidity index	0	(ref)		(ref)	
	1	0.92 (0.82–1.03)	0.13	0.91 (0.80–1.02)	0.12
	≥2	0.88 (0.77–0.99)	0.04	0.83 (0.72–0.95)	0.009
African‐American race		1.80 (1.62–2.01)	<0.001	1.64 (1.47–1.83)	<0.001
Brachytherapy boost		0.68 (0.51–0.90)	0.008	1.06 (0.81–1.41)	0.66
Body mass index (per 5 kg/m^2^)		1.06 (1.02–1.10)	0.003	0.99 (0.95–1.03)	0.54
Year of diagnosis (per year)		1.02 (1.01–1.04)	0.003	1.02 (1.01–1.04)	<0.001
Median income (per $10 000)		0.99 (0.97–1.03)	0.88	0.99 (0.96–1.03)	0.74
Zip code percent with high school diploma (per 10%)		0.97 (0.90–1.04)	0.36	0.98 (0.91–1.06)	0.68

Abbreviations: CI, confidence interval; OR, odds ratio; PSA, prostate‐specific antigen; RT, radiation therapy.

**FIGURE 2:**
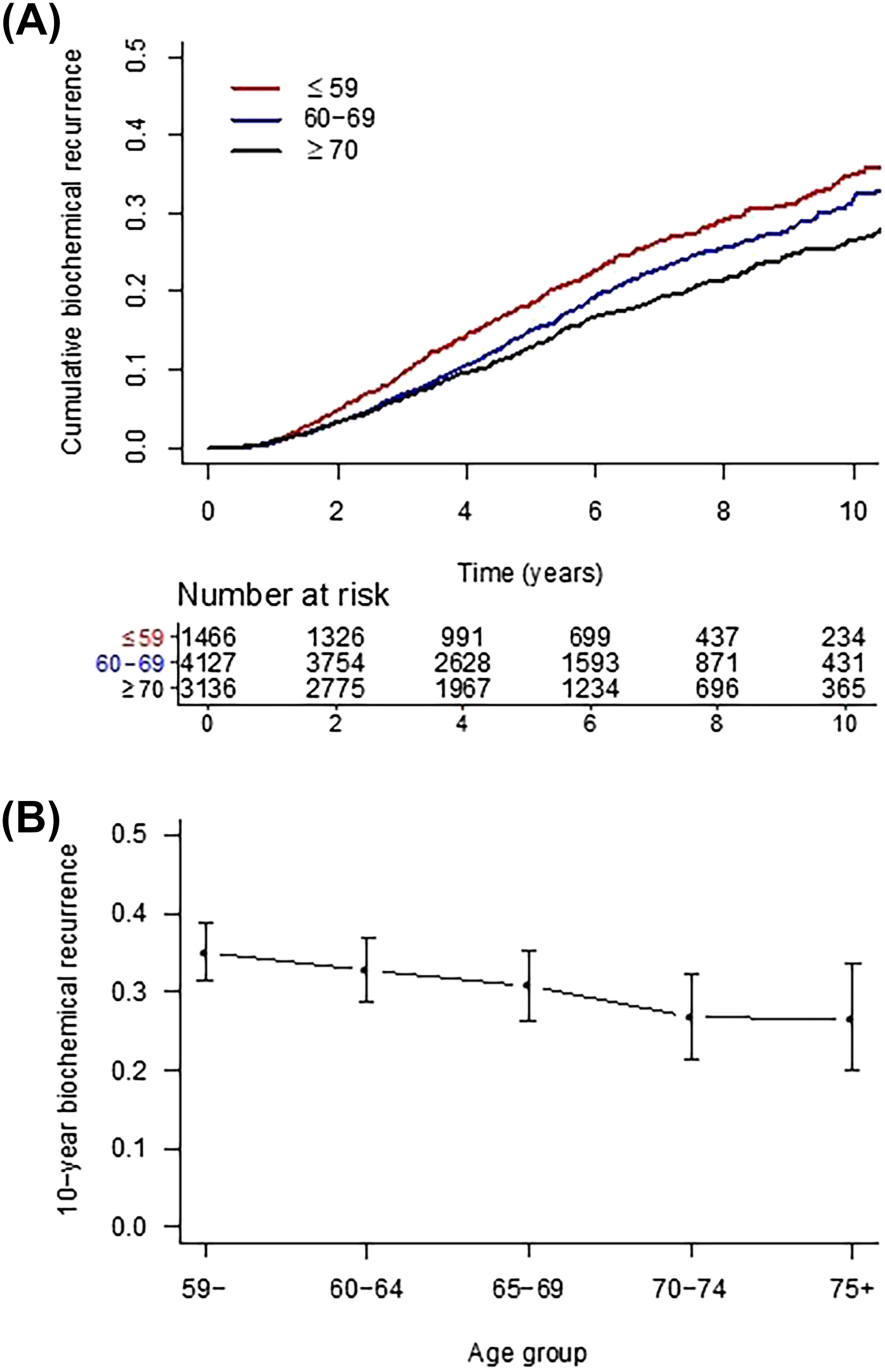
Biochemical recurrence by age at diagnosis. Panel (A) shows cumulative biochemical recurrence by age group; panel (B) plots actuarial 10‐year biochemical recurrence rates by age group with 95% confidence intervals

### 3.3 Effect of age on biochemical recurrence and survival

Median follow‐up for the sample was 6.3 years. Younger patients showed higher 10‐year rates of biochemical recurrence (≤59: 35.0% [95% CI 31.7%–38.4%]; 60–69: 31.8% [95% CI 29.5%–34.1%]; ≥70: 26.7% [95% CI 24.2%–29.1%], unadjusted *p* < 0.001 by log‐rank test; Figure 2). These differences persisted after multivariable adjustment in the Cox model (≤59 vs. ≥70: HR 1.45 [95% CI 1.26–1.67], *p* < 0.001; ≤59 vs. 60–69: HR 1.28 [95% CI 1.14–1.44], *p* < 0.001; Table 3). The cumulative incidence of PCSM was similar across age groups (≤59: 8.00% [95% CI 6.50%–9.47%]; 60–69: 6.90% [95% CI 6.00%–7.80%]; ≥70: 8.53% [95% CI 7.52%–9.53%], unadjusted *p* = 0.10 by Gray's test). There remained no difference between age groups after multivariable adjustment (Table 3). In sensitivity analyses, we repeated the above analyses for PSA response, biochemical recurrence, and PCSM among the intermediate risk patient subgroup, and similar results were observed (Table S1).

**TABLE 3:** Regression results for biochemical recurrence and prostate cancer‐specific mortality

Covariate		Biochemical recurrence		PCSM	
		OR (95% CI)	*p* value	SDHR (95% CI)	*p* value
Age group	≥70	(ref)		(ref)	
	60–69	1.28 (1.14–1.44)	<0.001	1.01 (0.85–1.19)	0.95
	≤59	1.45 (1.26–1.67)	<0.001	1.17 (0.94–1.46)	0.16
Clinical tumour stage	1C‐2A	(ref)		(ref)	
	2B‐2C	1.30 (1.17–1.46)	<0.001	1.47 (1.23–1.76)	<0.001
	3	1.55 (1.30–1.84)	<0.001	1.96 (1.55–2.49)	<0.001
Gleason score	6	(ref)		(ref)	
	7	1.15 (0.93–1.44)	0.21	1.08 (0.76–1.52)	0.68
	8–10	1.53 (1.22–1.91)	<0.001	2.04 (1.44–2.89)	<0.001
ln (pre‐treatment PSA)		1.68 (1.58–1.78)	<0.001	1.25 (1.14–1.38)	<0.001
Anti‐androgen therapy		0.94 (0.85–1.04)	0.24	1.10 (0.95–1.29)	0.22
Charlson comorbidity index	0	(ref)		(ref)	
	1	0.96 (0.86–1.08)	0.59	1.00 (0.83–1.19)	0.96
	≥2	0.99 (0.86–1.14)	0.95	1.14 (0.93–1.39)	0.19
African‐American race		0.96 (0.86–1.07)	0.47	0.89 (0.75–1.06)	0.20
Brachytherapy boost		0.64 (0.46–0.87)	0.005	0.71 (0.42–1.21)	0.21
Body mass index (per 5 kg/m^2^)		0.96 (0.92–1.00)	0.06	0.78 (0.72–0.84)	<0.001
Year of diagnosis (per year)		1.01 (1.00–1.03)	0.09	0.95 (0.93–0.97)	<0.001
Median income (per $10 000)		1.01 (0.98–1.04)	0.64	1.00 (0.96–1.05)	0.88
Zip code percent with high school diploma (per 10%)		0.98 (0.92–1.05)	0.53	0.95 (0.85–1.06)	0.35

Abbreviations: CI, confidence interval; OR, odds ratio; PCSM, prostate cancer‐specific mortality; PSA, prostate‐specific antigen; SDHR, subdistribution hazard ratio.

## 4 DISCUSSION

In this observational cohort study of more than 12 000 US veterans with localized prostate cancer treated with ADT and RT, we found that younger age at diagnosis was associated with inferior 3‐month post‐RT PSA response, higher 2‐year PSA nadir, and higher risk of biochemical recurrence, though we noted no effect on PCSM in the competing risk analysis. These differences remained significant after adjustment for multiple disease characteristics and demographic factors, and were robust in subgroup analyses of intermediate risk patients.

Our results suggest that younger patients may have a more aggressive disease course compared to older patients despite adjustment for baseline prognostic factors, translating to inferior immediate and long‐term PSA responses. Echoing these results, the recently proposed STAR‐CAP staging system includes young age (<50 years) as a negative prognostic factor for PCSM.^10^ This raises the question of whether younger patients with intermediate or high risk disease should consider treatment escalation beyond ADT and RT. Trials have demonstrated that adjuvant therapies such as abiraterone^20^ and brachytherapy boost^21^ improve biochemical progression‐free survival, and multimodality therapy combining surgery with RT and ADT may also improve outcomes in younger patients.^22^, ^23^ Treatment escalation may be especially beneficial in younger patients who carry lower competing mortality risks.^24^, ^25^

Our finding of a higher rate of biochemical recurrence in younger men is consistent with a previous multi‐institutional study of post‐RT PSA dynamics by Proust‐Lima et al.^7^ Their analysis of 4247 patients suggested that younger age was associated with a higher risk of biochemical recurrence, though the authors noted no effect on short‐term post‐treatment PSA dynamics. Previous studies on smaller cohorts treated with RT^11^, ^12^, ^13^ or prostatectomy^26^ have suggested no differences in biochemical recurrence, though these studies may have been underpowered to detect a significant difference in outcomes. Unique strengths of our study included the size of the Veterans Affairs database and the availability of longitudinal PSA measurements, allowing greater statistical power to detect long‐term differences in biochemical recurrence.

In addition to biochemical recurrence, we describe age‐based differences in short‐term PSA response and post‐treatment PSA nadir, both of which have been repeatedly demonstrated to impart early prognostic information and reflect successful treatment response.^15^, ^17^ This raises the possibility that younger age adversely affects the response to treatment, and that this effect is detectable within 3 months after completion of RT. Differences in treatment aggressiveness are unlikely to account for this result, as we did not observe significant differences in ADT type or duration across age groups and all patients were treated with definitive RT. The potential biologic mechanism of this effect is unknown and merits further investigation; possibilities include inadequate suppression of serum androgens by ADT or a higher proportion of androgen insensitive tumours in younger men. Some authors have suggested that tumours in younger men exhibit a particularly aggressive genetic phenotype, which may partly explain our findings^27^; however, further study is needed to delineate the specific molecular changes that may drive aggressive tumours in young men.

Our study is subject to several limitations. First, longitudinal PSA data were available in only a subset of the sample (Figure 1), and the exclusion of patients with missing PSA data may introduce bias. Our cohort was comprised exclusively of US veterans who may systematically differ from the general population of US men; this could decrease generalizability, and our results should be replicated in a sample of patients more representative of the US population. While we focused on intermediate‐ and high‐risk patients treated with ADT and RT, these results may not generalize to the broader population of patients with low‐risk disease, patients treated with definitive RT alone, or surgically managed patients. In particular, as younger men are preferentially treated with radical prostatectomy in many practices,^28^ younger men treated with radiation in our study may be subject to selection bias. As patients may seek follow‐up care outside of the VA system, we may systematically under‐ascertain biochemical recurrence. We noted a much higher proportion of African‐American men in the younger age groups (53% in ≤59 years vs. 24% in ≥70 years). While it has been suggested that African‐American men may have more aggressive disease phenotypes,^29^ others have suggested no differences in outcomes among men treated in equal‐access health care systems,^30^, ^31^ and as such, this is unlikely to bias our findings. RT dose was not available, though it is unlikely that younger men would be preferentially treated with lower radiation doses that would confound our results. Finally, though we observed similar age distributions throughout the years of the study period, there have been large changes in RT technique over time that may affect our results. Though we adjust for year of diagnosis in our models to account for time‐trends, it is possible that additional unmeasured confounding exists.

In summary, in this study of US veterans with localized prostate cancer treated with upfront ADT and RT, we demonstrate that younger age is associated with inferior short‐term PSA responses and higher rates of biochemical recurrence. The underlying mechanisms linking younger age and inferior treatment response are currently unknown and deserve further study.

## CONFLICT OF INTEREST

The authors declare no conflicts of interest.

## Supporting information


**Table S1.** Outcomes of primary analyses among intermediate risk patients.
