# Supplementary material for: Impact of age on treatment response in men with prostate cancer treated with radiotherapy
Source: BJUI Compass. 2021 Dec 27;3(3):243–50. doi: 10.1002/bco2.132 (PMC9045578; doi:10.1002/bco2.132)
Supplement: Supplementary file 1 — Table S1. Outcomes of primary analyses among intermediate risk patients. [file BCO2-3-243-s001.docx]

**Supplemental Table 1.** Outcomes of primary analyses among intermediate risk patients.

| **Outcome** | **Age group** | **OR, HR, or SDHR (95% CI)^1^** | **P-value** |
| --- | --- | --- | --- |
| 3 month post-RT PSA | ≥ 70 | (ref) | (ref) |
|  | 60-69 | 1.41 (1.18-1.68) | <0.001 |
|  | ≤ 59 | 2.07 (1.66-2.58) | <0.001 |
| 2-year PSA nadir | ≥ 70 | (ref) | (ref) |
|  | 60-69 | 1.45 (1.21-1.74) | <0.001 |
|  | ≤ 59 | 2.26 (1.82-2.80) | <0.001 |
| Biochemical recurrence | ≥ 70 | (ref) | (ref) |
|  | 60-69 | 1.32 (1.08-1.62) | 0.007 |
|  | ≤ 59 | 1.59 (1.25-2.02) | <0.001 |
| PCSM | ≥ 70 | (ref) | (ref) |
|  | 60-69 | 0.88 (0.65-1.19) | 0.40 |
|  | ≤ 59 | 1.02 (0.68-1.53) | 0.92 |

1: Odds ratios reported for the outcomes of 3-month post-RT PSA and 2-year PSA nadir. Hazard ratios reported for biochemical recurrence. Subdistribution hazard ratios reported for prostate cancer-specific mortality.

OR: odds ratio; HR: hazard ratio; SDHR: subdistribution hazard ratio; RT: radiotherapy; PSA: prostate specific antigen; PCSM: prostate cancer specific mortality.
